# Chronic Pruritus Severity and Its Association with Clinical Frailty in Geriatric Dermatology Patients: A Cross-Sectional Study

**DOI:** 10.3390/jcm14248809

**Published:** 2025-12-12

**Authors:** Gökçe Işıl Kurmuş, Dilek Menteşoğlu, Süheyla Çöteli, Selda Pelin Kartal

**Affiliations:** 1 Department of Dermatology & Venereology, Ankara Etlik City Hospital, Ankara 06170, Turkey; 2 Department of Geriatric Medicine, Ankara Etlik City Hospital, Ankara 06170, Turkey

**Keywords:** chronic pruritus, frailty, xerosis, FRAIL scale, polypharmacy, geriatric dermatology, overall dryness score

## Abstract

**Background/Objectives:** Chronic pruritus is a common and distressing symptom in geriatric dermatology, often intensified by age-related changes and frailty. Frailty, reflecting physiological decline and reduced resilience, may increase itch perception, yet its link to pruritus severity remains unclear. Our study aims to evaluate the association between pruritus severity and frailty in older dermatology patients and identify related clinical and dermatologic factors. **Methods:** A total of 171 patients aged ≥65 years with pruritus lasting more than six weeks were included. Pruritus was measured using the Visual Analog Scale (VAS) and 5-D Itch Scale, while frailty was assessed by the FRAIL scale. Xerosis severity was rated by the Overall Dryness Score (ODS), and data on itch duration, comorbidities, and medications were analyzed. Statistical significance was defined as *p* < 0.05. **Results:** The mean VAS and 5-D Itch scores were 6.2 ± 2.6 and 12.5 ± 3.1. Itch severity increased significantly across frailty categories (*p* < 0.001). Strong correlations were found between VAS and itch duration (r = 0.79, *p* < 0.001), ODS (r = 0.66, *p* < 0.001), and medication count (r = 0.56, *p* < 0.001). Similar associations were seen for 5-D Itch (r = 0.61, 0.64, and 0.54; *p* < 0.001). Age and comorbidities showed no significant correlation (*p* > 0.05). **Conclusions:** Pruritus severity in older adults was associated with xerosis, frailty, and polypharmacy, while no significant association with age was observed in univariable analyses. Incorporating frailty screening, ODS evaluation, and geriatric consultation into dermatologic care may improve management and quality of life in older adults with chronic pruritus. These findings reflect associations rather than causal relationships, as the cross-sectional design does not permit inference of directionality.

## 1. Introduction

Chronic pruritus, defined as itching persisting for more than six weeks, is one of the most frequent dermatological complaints in the geriatric population [1,2]. Its prevalence varies between 11% and 78%, with a tendency to increase with age [3]. Chronic itch not only causes persistent discomfort but also leads to sleep disturbance, depression, and a marked reduction in quality of life [4]. As populations age worldwide, chronic pruritus has emerged as a significant clinical and psychosocial burden in geriatric dermatology [5,6,7].

The aging process induces structural and functional changes in the skin that predispose older adults to chronic pruritus [8]. Age-related xerosis, decreased sebaceous activity, impaired epidermal barrier function, and pH alteration contribute to enhanced skin dryness and pruritus [9]. Immunosenescence, characterized by an imbalance of Th1/Th2 cytokine responses and increased interleukin-31 expression, promotes chronic inflammation and heightened sensitivity to pruritogens [1,9]. In addition, neuropathic mechanisms such as peripheral C-fiber hypersensitization and altered central itch processing further exacerbate itch perception in older adults [10]. These biological alterations, combined with environmental triggers, make the geriatric skin particularly vulnerable to persistent itch [1,9].

The etiology of chronic pruritus in older adults is often multifactorial, including dermatologic disorders such as xerosis, systemic diseases like renal or hepatic failure, and psychogenic or drug-induced causes [9,10,11,12,13]. Among these, xerosis remains the most prevalent factor in a study, observed in up to 68.9% of affected individuals [4].

Frailty, a multidimensional geriatric syndrome, reflects reduced physiological reserve, systemic inflammation, and vulnerability to external stressors [14]. Recent evidence suggests that chronic low-grade inflammation, mitochondrial dysfunction, and cellular senescence play key roles in the biological basis of frailty [15]. It frequently coexists with polypharmacy and chronic inflammatory states that may intensify pruritus [16]. However, the potential link between pruritus severity and frailty has not been systematically studied in dermatological practice.

Chronic pruritus in older adults is thought to result from a multifactorial interaction of dermatologic, neurologic, metabolic and immunosenescent processes [7,8,9]. Frailty, reflecting reduced physiological reserve and increased vulnerability to stressors, has been suggested as a potentially relevant factor in geriatric symptom burden [14,15]. Recent studies indicate that frailty may be associated with altered sensory processing, increased susceptibility to xerosis and greater medication use, all of which have been linked to pruritus in older individuals [12,13,14,15,16]. However, the relationship between frailty and pruritus severity has not been comprehensively examined, and evidence integrating frailty with key dermatologic determinants remains limited. The present study aimed to investigate the association between chronic pruritus severity and clinical frailty in geriatric dermatology patients.

## 2. Materials and Methods

### 2.1. Study Design and Setting

This cross-sectional observational study was conducted between April 2023 and October 2023 in the dermatology outpatient clinic of Ankara Etlik City Hospital. Participants were recruited consecutively among geriatric patients aged 65 years and older who presented with pruritus lasting for more than six weeks, consistent with the diagnostic definition of chronic pruritus. Written informed consent was obtained from all participants or their legal representatives prior to enrollment. The study was approved by the Ankara Etlik City Hospital Ethics Committee (Approval no: AEŞH-EK1-2023-042, Approval Date: 5 April 2023). Patients with incomplete clinical, frailty, xerosis, or pruritus assessments were excluded from the analysis, and no imputation methods were used to handle missing data.

### 2.2. Inclusion and Exclusion Criteria

Patients aged 65 years and older with clinically confirmed chronic pruritus lasting longer than six weeks and attending the dermatology outpatient clinic were eligible for inclusion. All participants were required to provide written informed consent and to complete the questionnaires reliably.

Systemic comorbidities frequently observed in older adults, including hypertension, diabetes mellitus, chronic kidney disease, thyroid disorders, cardiovascular disease and anemia, were recorded based on medical history and previous evaluations. These conditions are frequently encountered in geriatric pruritus research and were documented as part of the clinical profile of each participant. Medication use was recorded in detail. Polypharmacy was defined as the regular use of five or more systemic medications. Only systemic medications were counted toward the polypharmacy definition; topical treatments, OTC creams, and emollients were excluded because they may directly influence xerosis or itch.

**Figure 1:**
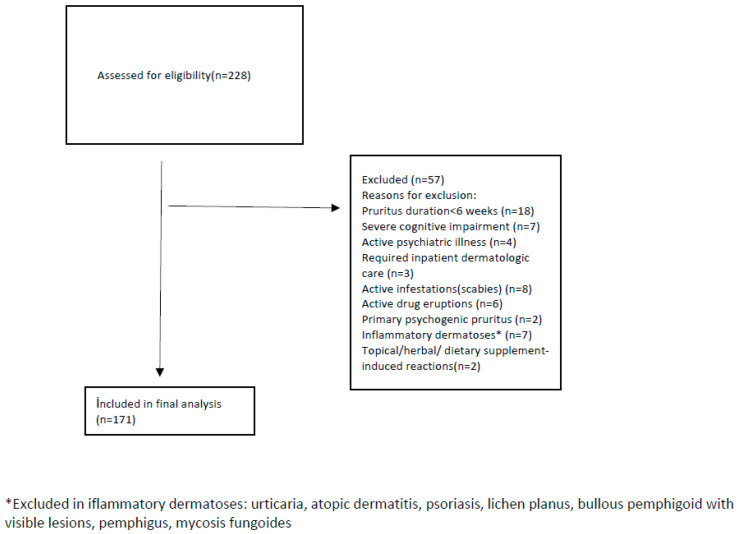
CONSORT-style flow diagram of study population.

Exclusion criteria focused on conditions that would interfere with accurate assessment or represent distinct causes of acute pruritus. These included pruritus lasting less than six weeks, severe cognitive impairment preventing completion of questionnaires, major psychiatric illness, need for inpatient dermatologic care, active infestations such as scabies, acute drug eruptions and primary psychogenic pruritus. Cognitive status was evaluated clinically, and patients were excluded only if impairment was severe enough to prevent reliable questionnaire completion. In addition, dermatologic diseases known to cause acute or chronic inflammatory pruritus were excluded. These included urticaria, atopic dermatitis, psoriasis, lichen planus, bullous pemphigoid with visible skin lesions, pemphigus, mycosis fungoides and other inflammatory dermatoses with active cutaneous findings. Patients whose pruritus began immediately after the use of topical products, herbal preparations or dietary supplements were also excluded, since these cases represent acute irritant or allergic reactions rather than chronic pruritus. These criteria ensured a consistent outpatient population suitable for evaluating pruritus severity, frailty and polypharmacy, as illustrated in the participant flow chart (Figure 1).

### 2.3. Data Collection

Demographic and clinical parameters were recorded for each participant. Variables included age, sex, duration of pruritus (months), number of comorbidities, number of medications, polypharmacy status, and xerosis severity. Polypharmacy was defined as the regular use of five or more medications. Xerosis was evaluated with the Overall Dry Skin (ODS) score, ranging from 0 (no dryness) to 4 (severe scaling or fissures).

### 2.4. Pruritus and Frailty Assessments

Pruritus severity was evaluated using a 10-point Visual Analog Scale (VAS) and the Turkish-validated version of the 5-D Itch Scale, which assesses five domains: duration, degree, direction, disability, and distribution [17]. Pruritus was confirmed through a standardized clinical dermatologic examination and patient-reported symptoms. To minimize inter-observer variability, all pruritus assessments, including VAS and 5-D Itch scoring, were performed by the same dermatologist during the outpatient visit. Frailty assessment was performed in collaboration with the Department of Geriatric Medicine to ensure consistency and accuracy. Frailty was measured using the validated FRAIL scale, a five-item geriatric screening tool evaluating fatigue, resistance, ambulation, illnesses, and unintentional weight loss. Each positive component was scored as one point (range: 0–5). Based on established criteria, participants were categorized as non-frail (0 points), pre-frail (1–2 points), or frail (≥3 points). All assessments were completed during the dermatology outpatient visit and verified by a geriatric medicine specialist.

### 2.5. Statistical Analysis

All statistical analyses were performed using SPSS Statistics version 22.0 (IBM Corp., Armonk, NY, USA). A priori power analysis using G*Power (version 3.1) indicated that at least 111 participants were required (effect size = 0.30, power = 0.80, α = 0.05). The final sample size of 171 patients was therefore adequate. Descriptive data were expressed as mean ± standard deviation (SD), median [interquartile range (IQR)], or frequency (percentage), as appropriate. The normality of data distribution was assessed using the Shapiro–Wilk test. Because the majority of variables did not meet the assumptions of normality, nonparametric methods were applied. The Kruskal–Wallis test was used to compare continuous variables among frailty categories, followed by Dunn–Bonferroni post hoc tests for pairwise comparisons. The Mann–Whitney U test was applied to assess differences between polypharmacy groups, while the chi-square test evaluated associations between categorical variables. Correlations between pruritus severity scores (VAS and 5-D Itch total) and clinical parameters were analyzed using Spearman’s rank correlation coefficient. A *p*-value < 0.05 was considered statistically significant.

## 3. Results

A total of 171 geriatric patients with chronic pruritus were evaluated. The mean age was 73.1 ± 6.5 years (median = 71.0, IQR = 68.0–77.5), and 55.6% were female (Table 1). The median duration of pruritus was 10 months [IQR = 7.0–15.5]. The mean number of medications was 4.4 ± 1.5, and polypharmacy (≥5 drugs) was identified in 48.5% of patients. The median number of comorbidities was 1.0 [IQR = 1.0–2.0], and the most common comorbidities were hypertension (40.5%), diabetes mellitus (25.0%), and cardiovascular disease (16.7%) (Table 1).

The mean VAS score for pruritus severity was 6.2 ± 2.6, and the mean 5-D Itch total score was 12.5 ± 3.1 (Table 1). According to xerosis grading, 42.7% of patients had no xerosis, 34.5% had mild, and 22.2% had moderate xerosis. Based on frailty assessment, 15.8% of participants were non-frail, 40.4% were pre-frail, and 43.9% were frail (Table 1).

**Figure 2:**
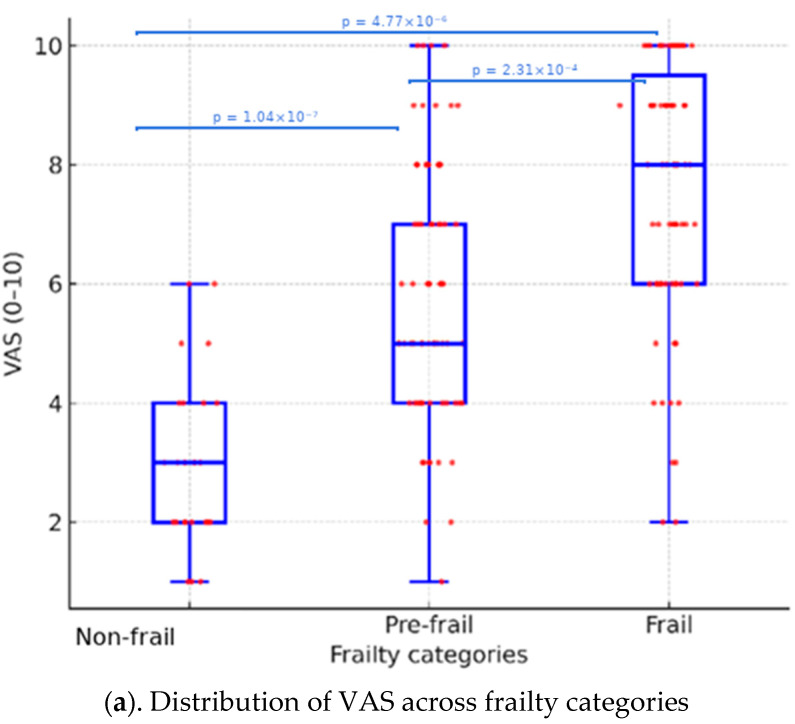

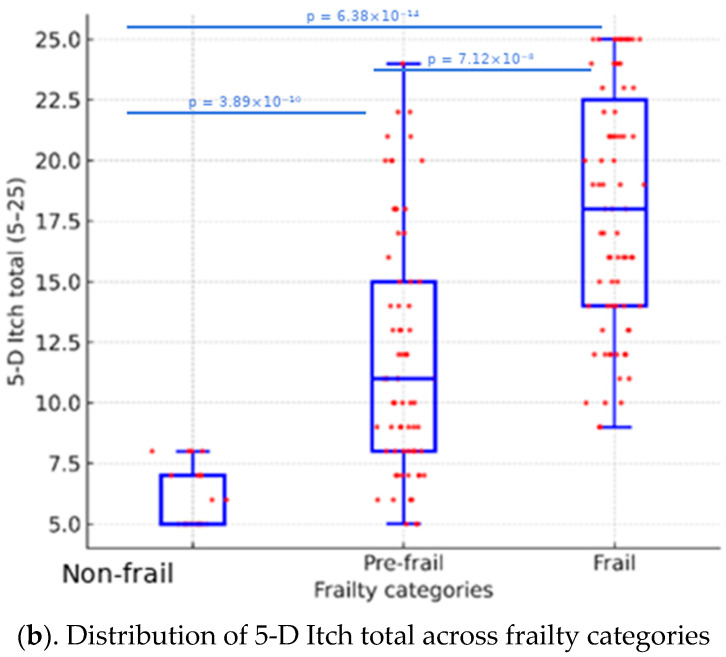
(**a**) Comparison of Visual Analog Scale (VAS) scores among frailty categories. Mean VAS values were significantly higher in frail patients compared with pre-frail and non-frail groups (*p* < 0.001, Kruskal–Wallis test). (**b**) Comparison of 5-D Itch total scores among frailty categories. The frail group showed markedly elevated 5-D Itch scores compared with pre-frail and non-frail groups (*p* < 0.001, Kruskal–Wallis test). Values are presented as mean ± SD and median [IQR]. Red dots represent individual patient data points.

Comparison of pruritus severity among frailty categories demonstrated a progressive increase in both VAS and 5-D Itch scores with rising frailty levels. The median VAS score was 3.0 [2.0–4.0] in non-frail, 5.0 [4.0–7.0] in pre-frail, and 8.0 [6.0–9.5] in frail patients (*p* < 0.001) (Table 2, Figure 2a). The median 5-D Itch total score increased from 5.0 [5.0–7.0] to 11.0 [8.0–15.0] and 18.0 [14.0–22.5], respectively (*p* < 0.001) (Table 2, Figure 2b). Post hoc analyses demonstrated significant differences between all frailty groups for both VAS and 5-D Itch scores, with exact adjusted *p*-values ranging from 1.04 × 10^−7^ to 4.77 × 10^−6^ for VAS and from 3.89 × 10^−10^ to 6.38 × 10^−14^ for 5-D Itch (Figure 2). Itch duration, number of comorbidities, polypharmacy, and xerosis severity were also significantly higher in the frail group (*p* ≤ 0.05) (Table 2).

In the subgroup analysis according to polypharmacy, patients with polypharmacy had higher median VAS (8.0 [6.0–9.5]) and 5-D Itch total scores (18.0 [13.0–21.5]) than those without polypharmacy (5.0 [3.0–6.0] and 9.0 [6.8–13.0], respectively; *p* < 0.001) (Table 3). They also had longer itch duration (13.0 [10.0–22.0] months) and more comorbidities (*p* < 0.001). Frailty prevalence was significantly greater among polypharmacy patients (60.2%) compared with non-polypharmacy participants (28.4%) (*p* < 0.001) (Table 3).

Pruritus severity showed no significant difference according to the number of comorbidities (*p* > 0.05) (Table 4). However, both VAS and 5-D scores increased progressively with higher xerosis grades (*p* < 0.001) (Table 5).

Correlation analysis revealed strong positive correlations between pruritus severity scores and itch duration (*r* = 0.786 and 0.610 for VAS and 5-D, respectively; *p* < 0.001), number of medications (*r* = 0.555 and 0.538; *p* < 0.001), and xerosis severity (*r* = 0.660; *p* < 0.001) (Table 6). A weak correlation was found between VAS and the number of comorbidities (*r* = 0.188; *p* = 0.014), while no significant correlation was observed with age (*p* > 0.05) (Table 6).

## 4. Discussion

This study analyzed 171 geriatric patients with chronic pruritus, with a mean age of 73.1 ± 6.5 years and a slight female predominance (55.6%) (Table 1). Comparable demographic distributions have been documented in previous studies, in which the mean age of participants ranged between 70 and 76 years, and the prevalence of pruritus was consistently higher among women [4,5,7]. The greater susceptibility among geriatric females may be related to postmenopausal hormonal decline, reduced epidermal lipid synthesis, and skin dryness, all of which impair the barrier function and enhance peripheral nerve sensitivity [13].

In our population, the median itch duration was 10 months [IQR 7.0–15.5], reflecting the persistent and relapsing nature of the condition (Table 1). In a recent study, Günther et al. observed that chronic pruritus affected 23.4% of geriatric individuals, with a mean duration of 8.4 years and 70.2% of patients experiencing symptoms for longer than one year [2]. Reszke et al. examined 153 geriatric patients and found that 35.3% had chronic pruritus, with nearly half (46.3%) describing itch episodes lasting longer than ten minutes [7]. Valdés-Rodríguez et al. reported that 55% of patients had symptoms lasting 12–36 months, and 20% for more than 36 months [4]. Chronic itch in geriatric patients is often sustained by structural and immunologic skin alterations, including decreased sebaceous and sweat gland activity, impaired epidermal lipid composition, and immune dysregulation due to immunosenescence [10,11]. These mechanisms disrupt the cutaneous barrier and enhance the release of pruritogenic cytokines such as interleukin-31, contributing to the chronicity observed in our cohort.

The mean VAS score of 6.2 ± 2.6 and 5-D Itch total of 12.5 ± 3.1 were observed in our study. In a Malaysian outpatient cohort, the mean itch intensity measured by the Numeric Rating Scale (NRS) was 6.0 (IQR 4–8), and more than half of the participants reported moderate-to-severe pruritus, a distribution closely resembling our findings [18]. These findings parallel results from large geriatric cohorts using standardized pruritus questionnaires. In the study by Mazan et al., 79.6% of geriatric participants reported pruritus, and the mean VAS and NRS scores indicated moderate-to-severe intensity (VAS 6.1 ± 2.2; NRS 6.3 ± 2.1) [19]. Valdés-Rodríguez et al. reported a mean itch intensity of 6 ± 2.1 (range 3–10) using a validated VASItch scale, with higher values among patients presenting scalp and arm involvement [4]. This level of severity has been associated with reduced quality of life, sleep disturbance, and depressive symptoms in prior studies [2,7,18]. From a dermatologic perspective, this reflects not only sensory hypersensitivity but also central sensitization processes that develop with aging, involving altered C-fiber signaling and decreased inhibitory neurotransmission [9,13].

Comorbidities were frequent in our cohort, particularly hypertension (40.5%), diabetes mellitus (25.0%), and cardiovascular disease (16.7%) (Table 1). However, despite their high frequency, the number of coexisting conditions did not show a significant association with itch severity on VAS or with 5-D Itch total (*p* > 0.05) (Table 4). Similarly, Yong et al. reported that hypertension (60.9%) and diabetes mellitus (56.5%) were among the most common systemic conditions in older adults with generalized pruritus, although neither showed a significant association with itch presence [18]. In the study by Valdés-Rodríguez et al., hypertension was present in 44% of patients with chronic pruritus, followed by diabetes mellitus (35%), dyslipidemia (28%), chronic kidney disease (19%), and liver disease (12%) [4]. Systemic comorbidities may contribute to the persistence of pruritus by promoting microvascular changes, metabolic dysregulation, and oxidative stress, all of which impair skin homeostasis [6,11]. In addition, xerosis was detected in 57.3% of our patients. In Valdés-Rodríguez et al., xerosis was documented in 68.9% of older adults with chronic itch versus 17.5% without itch (*p* < 0.001), highlighting a strong co-occurrence with pruritus [4]. Günther et al. likewise noted xerosis in 72.3% of their cohort and described it as the predominant dermatologic correlate of chronic pruritus [2]. In another study xerosis was detected in 82.9% of those with itch, and higher ODS scores were strongly correlated with both VAS (r = 0.67) and 10-PSS scores (r = 0.71) [19]. Age-related xerosis involves reduced natural moisturizing factors, epidermal thinning, and increased transepidermal water loss, features that are associated with persistent itch [3,8]. In our analysis, both the presence of xerosis and ODS score were significantly associated with greater itch intensity and impairment across all 5-D Itch domains, including duration, degree, direction, disability, and distribution (*p* < 0.001) (Table 5). This indicates that xerosis is strongly associated with both the onset and persistence of chronic pruritus by increasing both the sensory and functional burden of itch. Similar results have been reported in geriatric cohorts, where increasing skin dryness correlated with higher itch scores and sleep disturbance severity [2,7]. Structural barrier damage in xerotic skin facilitates the penetration of irritants and pruritogens, while diminished lipid content and altered hydration reduce the threshold for peripheral nerve activation [1,11]. Furthermore, reduced natural moisturizing factors and altered corneocyte cohesion observed in aging skin impair epidermal repair mechanisms, sustaining chronic itch responses [11,12,13]. Taken together, these data highlight xerosis as a major clinical factor associated with pruritus severity and itch-related disability in older adults.

Collectively, these findings confirm that chronic pruritus in geriatric dermatologic patients is a multifactorial condition influenced by aging-related skin changes, comorbidities, and neuroimmune dysregulation. The high symptom burden observed in our cohort mirrors previous epidemiologic data and underlines the need for a multidimensional evaluation that addresses both cutaneous and systemic contributors in this vulnerable population. Although patients with active inflammatory dermatoses were excluded from this study, it is important to recognize that prodromal bullous pemphigoid may present solely with pruritus in older adults, and this diagnostic consideration remains relevant in geriatric clinical practice.

In our cohort, frailty showed a significant association with pruritus severity. Both VAS and 5-D Itch scores increased progressively across frailty categories, with the highest median values observed in frail patients (*p* < 0.001) (Table 2). These findings indicate that individuals with lower functional capacity appear to report more pronounced itch severity. Similar observations have been described in geriatric populations where frailty was closely linked to chronic symptoms, systemic inflammation, and reduced quality of life [14,20]. As shown in Figure 2, itch severity increased steadily with higher frailty levels.

The biological mechanisms underlying this relationship are multifactorial. Frailty is characterized by chronic low-grade inflammation, mitochondrial dysfunction, impaired cellular repair and immune dysregulation—collectively referred to as inflammaging—which may contribute to enhanced itch sensitivity [20,21]. Elevated pro-inflammatory cytokines such as interleukin-6, tumor necrosis factor-α, and C-reactive protein have been identified as biomarkers of frailty and are known to sensitize peripheral C-fibers and promote pruritogenic mediator release in the skin [11,20,21]. Additionally, oxidative stress and nutritional deficiencies—frequently observed in frail geriatric individuals—may further impair epidermal barrier integrity and neurosensory balance, predisposing patients to chronic itch [20,21,22]. From a dermatologic perspective, this shared inflammatory environment may explain the greater itch burden observed in frail individuals. Moreover, evidence from dermatologic cohorts supports that chronic inflammatory skin diseases in older adults are often accompanied by high frailty scores and reduced recovery capacity, as demonstrated in the GERAS study, where 83% of geriatric patients with chronic leg ulcers were classified as frail [23].

Polypharmacy was common among our participants, with 48.5% using five or more medications (Table 3). Although the total number of drugs was not directly associated with VAS or 5-D Itch scores, a positive correlation was observed between polypharmacy and frailty severity (r = 0.571, *p* < 0.001). These findings align with previous studies reporting that polypharmacy may accelerate frailty progression through mechanisms such as altered pharmacokinetics, increased inflammatory load, and cumulative adverse effects [14,16,24]. In a Japanese outpatient cohort, polypharmacy was also identified as an independent predictor of moderate-to-severe pruritus, suggesting that extensive medication use may indirectly amplify itch burden through systemic vulnerability rather than through a specific drug effect [25]. From a clinical standpoint, reviewing medication profiles in frail geriatric patients with chronic pruritus is therefore essential to minimize potential aggravating factors and optimize overall treatment outcomes.

Beyond biological pathways, the clinical interplay between frailty and chronic pruritus appears to be bidirectional. Chronic pruritus is strongly associated with impaired quality of life, including sleep disturbance, reduced social functioning, and increased risk of depressive symptoms, as demonstrated in several geriatric cohorts [2,4,7]. Persistent itching has been associated with factors such as sleep disruption, fatigue, and reduced appetite, which overlap with components of frailty [14,20]. Conversely, frail individuals often exhibit reduced resilience to stress and slower tissue recovery, making them more susceptible to prolonged skin inflammation and sensory hypersensitivity [21]. Previous reports have emphasized that systemic inflammation and malnutrition, common in frail elders, correlate with elevated cytokine levels and muscle catabolism, mechanisms that may equally intensify chronic pruritus [26,27]. In this context, frailty and pruritus likely reinforce each other in a mutually reinforcing cycle, where functional decline amplifies itch perception, and persistent pruritus further deteriorates physiological reserve and overall well-being.

The consistent rise in both sensory (VAS) and multidimensional (5-D) itch scores with higher frailty grades in our study supports the hypothesis that systemic vulnerability and skin dysfunction coexist as components of a shared aging phenotype. Frailty appears to be associated with greater pruritus burden.

In our correlation analysis, the intensity of pruritus demonstrated the strongest positive associations with itch duration (VAS: r = 0.786; 5-D Itch: r = 0.610, *p* < 0.001 for both) and xerosis severity as measured by ODS score (VAS: r = 0.660; 5-D Itch: r = 0.642, *p* < 0.001) (Table 6). A moderate correlation was also observed with the number of medications used (VAS: r = 0.555; 5-D Itch: r = 0.538, *p* < 0.001), suggesting that polypharmacy may indirectly increase itch burden through systemic and dermatologic effects. In contrast, age and total number of comorbidities showed only weak or nonsignificant correlations with itch severity (*p* > 0.05). Consistent with our results, Mazan et al. found no significant correlation between age, gender, or most comorbidities and pruritus severity assessed by VAS, NRS, and 10-PSS scales, except for patients with anemia, who demonstrated higher 10-PSS scores (*p* = 0.026) (19). These findings indicate that, among geriatric individuals, chronic pruritus was associated with skin-related and treatment-related factors in univariable analyses, while no significant association with chronological age or comorbidity burden was observed.

From a clinical standpoint, the observed correlations underline that chronic pruritus in older adults is not an isolated dermatologic condition but rather a manifestation of broader frailty-related decline. As functional dependence increases, so does the subjective and objective burden of itch, reinforcing the need for integrated assessment of skin, systemic health, and psychosocial function. These findings align with previous reports highlighting that higher frailty and polypharmacy scores correlate with impaired quality of life and reduced recovery capacity in chronic skin disorders [18,24]. Incorporating frailty screening and medication review into dermatologic evaluation may therefore facilitate early identification of patients at risk for persistent or disabling pruritus and guide holistic management strategies. Future research should explore longitudinal changes in frailty and pruritus severity, evaluate biomarkers of skin barrier dysfunction and inflammation, and assess whether targeted frailty interventions such as nutritional support or physical activity can reduce the burden of pruritus in older adults.

## 5. Conclusions

Taken together, these results emphasize that chronic pruritus in older adults is a multifactorial condition associated with xerosis, frailty, and polypharmacy rather than chronological aging alone. This study has several limitations, including its single-center, cross-sectional design and the absence of objective assessments of inflammatory and skin barrier parameters, which may limit generalizability. Patients with severe cognitive impairment or extreme frailty may be under-represented in this study due to the exclusion criteria. In addition, possible referral bias should be considered, as the study population consisted solely of patients who presented to a dermatology outpatient clinic. Because only outpatient cases were included and no long-term follow-up was available, changes in pruritus severity or frailty status over time could not be assessed. Because all statistical analyses in this study were univariable, independent effects of the examined variables could not be established. Given the cross-sectional study design, these results indicate associations rather than causation, and longitudinal studies are required to clarify temporal relationships. Nevertheless, recognizing the interplay between itch severity and frailty highlights the importance of integrating frailty screening, medication review, and geriatric consultation into dermatologic evaluation to optimize patient care and improve quality of life in older adults with chronic pruritus.

## Figures and Tables

**Table 1:** Baseline characteristics of the study population.

Variable	n (%)	Mean ± SD/Median [IQR]
**Demographics**		
Age, years		73.1 ± 6.5, 71.0 [68.0–77.5]
Female	95 (55.6)	
Male	76 (44.4)	
**Education level**		
Primary school or below	21 (12.3)	
Middle school	67 (39.2)	
High school	69 (40.4)	
University or above	14 (8.2)	
**Marital status**		
Married	113 (66.1)	
Single	8 (4.7)	
Divorced	12 (7.0)	
Widowed	38 (22.2)	
**Living situation**		
With family	130 (76.0)	
Alone	27 (15.8)	
Nursing home/institutionalized	14 (8.2)	
**Clinical characteristics**		
Itch duration, months		12.5 ± 7.9, 10.0 [7.0–15.5]
Number of drugs		4.4 ± 1.5, 4.0 [3.0–5.0]
Polypharmacy (≥5 drugs), yes	83 (48.5)	
Number of comorbidities		1.3 ± 1.0, 1.0 [1.0–2.0]
**Comorbidities**		
Hypertension, yes	68 (40.5)	
Diabetes, yes	42 (25.0)	
Cardiovascular disease, yes	28 (16.7)	
Renal disease, yes	15 (8.9)	
Thyroid disease, yes	12 (7.1)	
Anemia, yes	20 (11.9)	
Malignancy, yes	10 (6.0)	
**Pruritus severity**		
VAS (0–10)		6.2 ± 2.6, 6.0 [4.0–8.5]
5-D Itch total (5–25)		12.5 ± 3.1, 12.0 [10.0–15.0]
**Xerosis (ODS)**		
None	73 (42.7)	
Mild	59 (34.5)	
Moderate	38 (22.2)	
Severe	1 (0.6)	
**FRAIL categories**		
Non-frail (0)	27 (15.8)	
Pre-frail (1–2)	69 (40.4)	
Frail (3–5)	75 (43.9)	

Values are presented as mean ± standard deviation (SD), median [interquartile range (IQR)], or number (n) (%) as appropriate. VAS: Visual Analog Scale; 5-D: 5-Dimensional Itch Scale; ODS: Overall Dry Skin score.

**Table 2:** Comparison of clinical characteristics and pruritus severity according to frailty categories.

Variable	Non-Frail (n = 27)	Pre-Frail (n = 69)	Frail (n = 75)	*p*-Value
VAS (0–10)	3.0 ± 1.4, 3.0 [2.0–4.0]	5.8 ± 2.2, 5.0 [4.0–7.0]	7.7 ± 2.2, 8.0 [6.0–9.5]	<0.001 *
5-D Itch total score (5–25)	5.8 ± 1.2, 5.0 [5.0–7.0]	11.9 ± 5.0, 11.0 [8.0–15.0]	18.0 ± 5.0, 18.0 [14.0–22.5]	<0.001 *
Itch duration, months	8.2 ± 4.3, 7.0 [5.5–9.0]	12.2 ± 6.9, 10.0 [8.0–16.0]	14.3 ± 9.1, 11.0 [8.0–18.5]	0.001 *
Number of comorbidities	1.0 ± 0.9, 1.0 [0.0–2.0]	1.6 ± 1.0, 1.0 [1.0–2.0]	1.2 ± 1.0, 1.0 [0.5–2.0]	0.038 *
Polypharmacy, yes	4 (14.8%)	30 (43.5%)	49 (65.3%)	<0.001 *
Xerosis (ODS ≥ 2), yes	3 (11.1%)	17 (24.6%)	19 (25.3%)	0.041 *

Values are presented as mean ± standard deviation, median [interquartile range], or number (n) (%) as appropriate. *p*-values were obtained using the Kruskal–Wallis test for continuous variables and the chi-square test for categorical variables. * *p* < 0.05 was considered statistically significant. VAS: Visual Analog Scale; 5-D: 5-Dimensional Itch Scale; ODS: Overall Dry Skin score, ODS score ≥ 2 indicates moderate-to-severe xerosis.

**Table 3:** Comparison of clinical characteristics and pruritus severity according to polypharmacy status.

Variable	No Polypharmacy (n = 88)	Polypharmacy (n = 83)	*p*-Value
VAS (0–10)	4.8 ± 2.2, 5.0 [3.0–6.0]	7.7 ± 2.1, 8.0 [6.0–9.5]	<0.001 *
5-D Itch total (5–25)	10.3 ± 4.9, 9.0 [6.8–13.0]	17.2 ± 5.7, 18.0 [13.0–21.5]	<0.001 *
Itch duration, months	9.0 ± 5.6, 8.0 [6.0–10.0]	16.3 ± 8.3, 13.0 [10.0–22.0]	<0.001 *
Number of comorbidities	0.9 ± 0.8, 1.0 [0.0–1.2]	1.8 ± 1.0, 2.0 [1.0–3.0]	<0.001 *
Xerosis (ODS ≥ 2), yes	11 (12.5%)	28 (33.7%)	0.002 *
Frailty categories			<0.001 *
Non-frail	22 (25.0%)	5 (6.0%)	
Pre-frail	41 (46.6%)	28 (33.7%)	
Frail	25 (28.4%)	50 (60.2%)	

Values are presented as mean ± standard deviation, median [interquartile range], or number (n) (%). *p*-values were obtained using Mann–Whitney U test for continuous variables and chi-square test for categorical variables. * *p* < 0.05 was considered statistically significant. VAS: Visual Analog Scale; 5-D: 5-Dimensional Itch Scale; ODS: Overall Dry Skin score.

**Table 4:** Comparison of pruritus severity according to number of comorbidities.

Variable	0 Comorbidities (n = 37)	1 Comorbidity (n = 66)	≥2 Comorbidities (n = 68)	*p*-Value
VAS (0–10)	5.0 [3.0–7.0]	6.0 [5.0–9.0]	6.0 [4.0–9.0]	0.063
5-D Itch total (5–25)	10.0 [8.0–15.0]	14.0 [8.5–20.0]	12.0 [8.0–20.0]	0.097

Values are presented as median [interquartile range]. *p*-values were calculated using the Kruskal–Wallis test.

**Table 5:** Pruritus severity scores according to xerosis categories (ODS 0–4).

Variable	ODS 0—None (n = 73)	ODS 1—Mild (n = 59)	ODS 2—Moderate (n = 38)	ODS 3—Severe (n = 1)	*p*-Value
VAS (0–10)	5.0 [3.0–7.0]	6.0 [4.0–8.0]	7.0 [5.0–9.0]	9.0 [9.0–9.0]	<0.001 *
5-D Duration	2.0 [1.0–3.0]	3.0 [2.0–4.0]	3.0 [2.0–4.0]	4.0 [4.0–4.0]	<0.001 *
5-D Intensity	2.0 [1.0–3.0]	3.0 [2.0–4.0]	4.0 [3.0–5.0]	4.0 [4.0–4.0]	<0.001 *
5-D Direction	1.0 [1.0–2.0]	2.0 [1.0–2.0]	2.0 [2.0–3.0]	3.0 [3.0–3.0]	0.014 *
5-D Disability	2.0 [1.0–3.0]	3.0 [2.0–4.0]	3.0 [2.0–4.0]	4.0 [4.0–4.0]	<0.001 *
5-D Distribution	2.0 [1.0–3.0]	3.0 [2.0–4.0]	3.0 [2.0–4.0]	4.0 [4.0–4.0]	<0.001 *

Values are presented as median [interquartile range]. *p*-values were calculated using the Kruskal–Wallis test. Post hoc comparisons (Dunn–Bonferroni) indicated that higher xerosis categories were associated with significantly higher VAS and 5-D sub-scores (all *p* < 0.05). * *p* < 0.05 was considered statistically significant

**Table 6:** Correlation between pruritus severity and clinical parameters.

Variable	VAS (r, *p*)	5-D Itch Total (r, *p*)
Age	0.035 (*p* = 0.646)	0.052 (*p* = 0.503)
Itch duration (months)	0.786 (*p* < 0.001)	0.610 (*p* < 0.001)
Number of comorbidities	0.188 (*p* = 0.014 *)	0.111 (*p* = 0.148)
Number of medications	0.555 (*p* < 0.001)	0.538 (*p* < 0.001)
Xerosis (ODS score)	0.660 (*p* < 0.001)	0.538 (*p* < 0.001)

Correlation coefficients (r) and *p*-values were calculated using Spearman’s rank correlation test. * *p* < 0.05 was considered statistically significant.

## Data Availability

The original contributions presented in this study are included in the article. Further inquiries can be directed to the corresponding author.
